# CT and MRI Findings of Renal Angiomyolipoma With Lung Metastasis: A Case Report and Literature Review

**DOI:** 10.1111/crj.13796

**Published:** 2024-07-09

**Authors:** Zhiqiang He, Jing Wu, Xiao ming Fu, Xiao ran Li, Hai Xu, Yu‐Chen Chen

**Affiliations:** ^1^ Department of Radiology Nanjing Gaochun People's Hospital Nanjing China; ^2^ Department of Radiology Nanjing First Hospital, Nanjing Medical University Nanjing China; ^3^ Department of Radiology The First Affiliated Hospital of Nanjing Medical University Nanjing China

**Keywords:** CT, lung metastases, MRI, renal angioleiomyoma

## Abstract

Renal angiomyolipoma has two histological variants: classical and epithelioid. Epithelioid angiomyolipoma is considered as a potential malignant tumor, often leading to recurrence and metastasis, with rapid progression in most of the cases. The lung is one of the most commonly reported sites of metastasis, and pulmonary metastasis of renal angiomyolipoma is usually diagnostic by computed tomography (CT) scans. Here, we report for the first time renal angiomyolipoma with lung metastasis by combining CT and magnetic resonance imaging (MRI).

## Graphical abstract

We report for the first time renal epithelioid angiomyolipoma with lung metastasis by combining CT and magnetic resonance imaging (MRI).

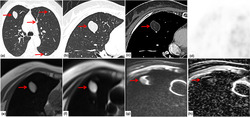

## 1 Introduction

Epithelioid angiomyolipoma (EASL) is a rare underlying malignancy, and once diagnosed, regular follow‐up imaging is required to detect recurrence or metastasis. We reported a 58‐year‐old female patient who was pathologically diagnosed with renal angiomyolipoma lung metastasis due to multiple nodule lesions in both lungs on physical examination computed tomography (CT). The patient has a history of left renal hamartoma resection. In this case, the patient had postoperative recurrence of angiomyolipoma with metastases in both lungs. The completed imaging data were reported, including nonenhanced and enhanced CT and magnetic resonance imaging (MRI). We reviewed previous literatures to provide a reliable reference for clinical diagnosis.

## 2 Case Presentation

A 58‐year‐old female patient was admitted to the hospital due to a physical examination. Chest CT showed multiple pulmonary nodules in both lungs. In 2011, the patient underwent a left kidney angiomyolipoma resection surgery at an external hospital. Enhanced CT scan performed on June 23, 2021, revealed multiple round‐shaped nodules with clear boundaries, various sizes, and fatty components in both lungs. The larger one was about 20 mm × 15 mm in size, and there is no significant enhancement. Pulmonary blood vessel shadows were observed within the nodules. PET‐CT imaging did not indicate significant uptake in the lung nodules. On June 25, a 3.0 T MRI (Siemens Skyra) showed multiple nodules in both lungs with short T1 and long T2 signals. The enhanced scan curve displayed no obvious enhancement. Since fat components were detected within the nodules, the imaging diagnosis indicated that the multiple nodules in both lungs were likely metastatic nodules, possibly resulting from the recurrence and metastasis of the left kidney angiomyolipoma after the surgery.

A needle biopsy was performed on the right upper lobe nodule and the similar histological features to the previously resected renal epithelioid AML. Additionally, a follow‐up abdominal CT revealed changes after the left kidney angiomyolipoma resection, with an 8 mm renal smooth muscle tumor in the left kidney showing clear boundaries and mild inhomogeneous enhancement in the enhanced scan. A biopsy was performed on the right upper lobe nodule, and the pathology showed the similar histological features to the previously resected adrenal epithelioid AML.

## 3 Discussion

Angiomyolipoma is a rare type of kidney tumor, accounting for 2%–6.4% of renal tumors [1]. When the epithelial component is the predominant feature, it is referred to as EAML [2]. Purely epithelial morphology is observed in 1% of renal AML cases [3]. Renal EASL is a less common malignant potential tumor originating from the renal interstitium which is first reported by Mai, Perkins, and Collins [4]. The incidence of metastatic disease in epithelioid AML is 5% [5]. It can occur in the lymph nodes, liver, spleen, lungs, uterus, heart, bones, brain, or in the retroperitoneum outside the kidney [6]. In previous reports, the disease progression was relatively rapid and many cases resulted in death within 2 years after recurrence or metastasis [5, 7, 8]. He et al. described a series of 437 renal AMLs. An epithelioid component was found in 20 cases (4.6%), and one (5%) of these patients developed metastatic disease after a mean follow‐up of 82.5 months [9]. All the patients with EAML had symptoms; none of them was incidentally discovered, a finding that could be explained in terms of aggressiveness [10].

There are few large institution‐based studies on EAML; vast majority of data have been documented in the literature principally as isolated case reports [1, 11, 12, 13, 14, 15, 16]. We searched previous cases about pulmonary metastatic renal angiomyolipoma from PubMed as listed in Table 1. In summary, a total of eight patients with pulmonary metastatic renal angiomyolipoma were reported. However, there was no specific literature describing the MRI characteristics of intrapulmonary lesions, and CT scans were rarely mentioned, while these data could provide important instructions for diagnosis of this diseases in the future. This highlights the important role of immunohistochemistry in the diagnosis of this disease.

**TABLE 1:** A compilation of case reports on pulmonary metastatic renal angiomyolipoma sourced from PubMed.

Studies	Age	Sex	Recurrence time (years)	Lung CT	Pathological immunohistochemistry
Martinioni, G	50	F	7	Isolated nodules, 2 cm	Epithelial HMB45 and A103+
Takahashi, N	44	M	3.3	—	HMB‐45 positive
	40	F	1.5	—	HMB‐45 and CD68 are strongly positive
Sato, K	36	M	2	—	HMB‐45 positive
Li Ji	55	F	7	The right lung is multiple, 1.5 cm	Melanoma‐related marker (HMB‐45) staining is strong
Shigenobu, T	46	F	7	Isolated nodules, 2 cm	HMB‐45+ S‐100 protein
MacCraith, E	68	F	4	Isolated, 1.6 cm	Positive for vimentin; CK7, S100, and CD 117 are negative
Sironi, M	61	F	14	Isolated, 4 cm	—

**FIGURE 1:**
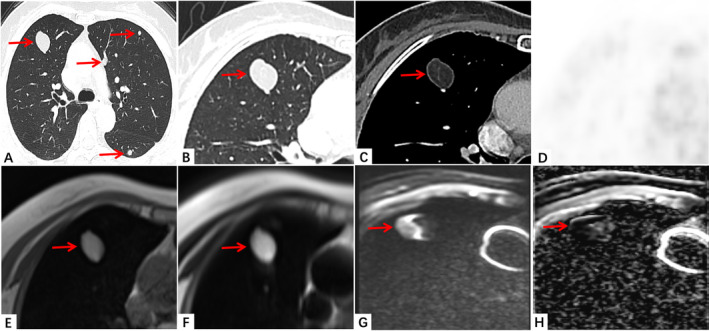
(A–H) The patient had CT MRI and PET‐CT examination. (A) Multiple metastatic nodules in both lungs. (B, C) The large nodular foci in the upper lobe of the right lung. CT contrast focus scan of nodules shows fat and vascular. The mean CT Value of this nodule was −91 HU. (D) No obvious uptake in PET‐CT at the same site. (E) The nodules are T1 high signal. (F) T2 high signal in MRI. (G) DWI high signal. (H) ADC low signal, diffusion is limited. The red arrows represented the lung nodules.

**FIGURE 2:**
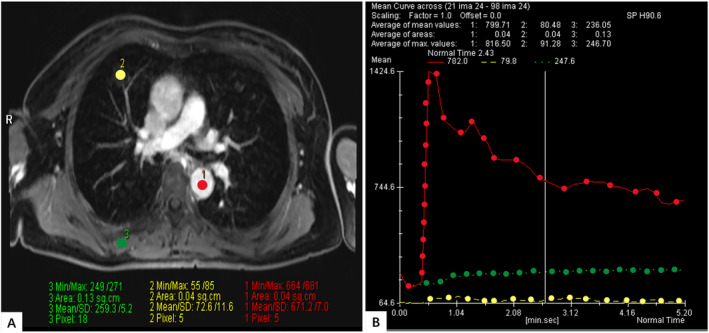
(A, B) The nodular enhancement scan. Red line represents the aortic enhancement curve. Yellow line represents the enhancement curve of lung metastasis nodules. The green line represents the curve of muscle tissue enhancement in the right back.

In the case we reported here who had a history of EAML 10 years ago, multiple nodules were detected in both lungs in the patient by a chest CT scan. These nodules were randomly distributed and had a relatively regular morphology, clear borders, and smooth edges. They contained fatty components and caused compression changes in peripheral bronchial compression, distinguishing them from other types of pulmonary metastatic tumors. However, in some instances, the fatty components within the lung lesion were not easily observed or even missed on CT image. Therefore, MRI was used to further confirm the presence of fat and vascular components within the lung nodules. As shown in Figures 1 and 2, on MRI, the lung lesions appeared with a regular morphology, exhibiting high signal intensity on both T1‐ and T2‐weighted images and low signal intensity on fat suppression sequences, indicating the presence of fatty components. After dynamic contrast‐enhanced scanning, no significant enhancement was observed within the lung lesions. Combined the findings from CT and MRI, the lung lesions are consistent with characteristics of metastatic nodules. Considering the presence of fat and vascular components within these lesions and also taking into account the patient's surgical history, these lung lesions are likely originating from angiomyolipoma of the kidney.

**FIGURE 3:**
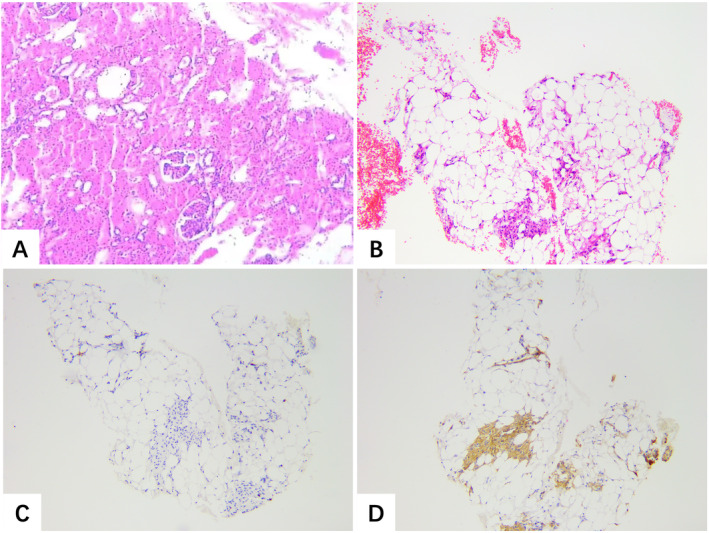
Pathological manifestations of patients after renal surgery and puncture of lung metastases. (A) The renal pathology is angiomyolipoma (hematoxylin and eosin staining of the nodule, original magnification × 40; scale bar = 50 μm). (B) The pathology of HE stained lung nodules is angiomyolipoma (hematoxylin and eosin staining of the nodule, original magnification × 200; scale bar = 50 μm). Immunohistochemistry results of panels (C, D) are SMA+, HMB45+, and Ki67 approximately 1% positive (hematoxylin and eosin staining of the nodule, original magnification × 200; scale bar = 50 μm).

By immunohistochemical assay, EAML is positive for melanocytic markers such as HMB‐45, melana protein and melanoma antigen recognized by T cells 1 (MART‐1), tyrosinase, and microphthalmia transcription factor but negative for epithelial markers and S‐100 protein [17, 18, 19, 20]. As shown in Figure 3, immunohistochemistry results showed SMA+, HMB45+, and Ki67 approximately 1% positive, which led to a definitive diagnosis of pulmonary metastatic epithelioid renal angiomyolipoma.

Primary EAML tumors after surgical resection have a risk of recurrence and distant metastasis, and if malignantly transformed, they often progress rapidly. Combining imaging features of lung nodules and immunohistochemistry results can provide valuable insights into the origin and nature of the lesions and is crucial in guiding the clinical diagnosis of this disease.

Therefore, postoperative patients should undergo regular monitoring with imaging studies to detect any signs of recurrence or metastasis. MRI is superior in displaying soft tissue signals, offers multiplanar imaging, and is more sensitive in detecting and characterizing lesions. CT provides shorter examination time and lower cost, but it involves ionizing radiation. For patients with history of EAML, to reduce financial burdens on the patient, a CT scan could be first performed; if a lesion with uncertain components is detected, MRI should be used in combination to make a diagnosis.

## Author Contributions

Conception and design: Zhiqiang He and Jing Wu. Acquisition, analysis, and interpretation of the data: Xiao ran Li and Hai Xu. Drafting of the manuscript: Zhiqiang He, Jing Wu, and Yu‐Chen Chen. All authors reviewed and approved the final version of the manuscript. All authors had read and approved the manuscript.

## Ethics Statement

The authors are accountable for all aspects of the work in ensuring that questions related to the accuracy or integrity of any part of the work are appropriately investigated and resolved. Written informed consent was obtained from all participants before their participation in the study protocol. The current study was approved by the Institutional Review Board of Nanjing Medical University.

## Conflicts of Interest

The authors declare no conflicts of interest.

## Data Availability

Data sharing not applicable to this article as no datasets were generated or analysed during the current study.
